# Novel use of anifrolumab in a young patient with Jo-1-positive juvenile dermatomyositis

**DOI:** 10.1093/rap/rkag017

**Published:** 2026-03-05

**Authors:** Daniel Windschall, Tanja Hinze, Sven Hardt, Duygu Aydin, Faekah Gohar

**Affiliations:** Clinic for Paediatric and Adolescent Rheumatology, St. Josef-Stift Sendenhorst, Northwest-German Center for Rheumatology, Sendenhorst, Germany; Medical Faculty, University of Halle-Wittenberg, Halle, Germany; Clinic for Paediatric and Adolescent Rheumatology, St. Josef-Stift Sendenhorst, Northwest-German Center for Rheumatology, Sendenhorst, Germany; Clinic for Paediatric and Adolescent Rheumatology, St. Josef-Stift Sendenhorst, Northwest-German Center for Rheumatology, Sendenhorst, Germany; Department of Pediatric Rheumatology, Kocaeli University, Kocaeli, Turkey; Clinic for Paediatric and Adolescent Rheumatology, St. Josef-Stift Sendenhorst, Northwest-German Center for Rheumatology, Sendenhorst, Germany

Key messages

- Refractory Jo-1-positive juvenile dermatomyositis with calcinosis cutis shows favourable response to anifrolumab.

Dear Editor, Anifrolumab is a monoclonal antibody targeting subunit 1 of the type 1 interferon (IFN) receptor. Experience with anifrolumab in patients with JDM is limited to predominantly adult or adolescent patients treated with a higher dose (300 mg/month) for skin and muscle disease, without calcinosis cutis and without longitudinal IFN signature assessments reported [1–3]. Two patients with anti-transcription intermediary factor 1γ–positive JDM and rapid response to anifrolumab were published in June 2025 [4]. Anifrolumab is currently approved for the treatment of SLE in adults and a study of anifrolumab in paediatric SLE is currently under way.

This case report describes the changes in disease activity and expression of type 1 IFN-stimulated genes in whole blood over 7 months with off-label use of anifrolumab (150 mg/month) in an 8-year-old girl with refractory skin involvement combined with severe calcinosis cutis.

Jo-1-positive JDM was first diagnosed at the age of 4 years, with Gottron’s papules, skin erythema (Fig. 1C) and MRI-confirmed myositis present. The initial treatment included methylprednisolone pulse therapy, oral prednisolone and methotrexate. Six months later, hydroxychloroquine was added. The treatment was ineffective, leading to an increased oral prednisolone dose after 12 months of therapy. Off-label tofacitinib, initiated 16 months after diagnosis, with a starting dose of 3.75 mg twice daily, followed by an increase to 4 mg twice daily after 14 months of treatment, significantly improved myositis [Childhood Myositis Assessment Scale (CMAS): 37–51 (maximum 52)] and the 20-item DAS [DAS20: 15 to 9 (worst 20)]. Minimal change in the IFN signature (median of the whole blood expression of six type 1 IFN-stimulated genes measured by real-time PCR: *IFI27*, *IFI44L*, *IFIT1*, *ISG15*, *RSAD2*, *SIGLEC1*; normal <6.0, moderate activity 20.1—50.0) occurred (15.6 to 9.4). Skin involvement remained refractory and calcinosis cutis developed 3.5 years after first presentation. The addition of immunoglobulin therapy and switching from tofacitinib to baricitinib, administered at a dose of 2 mg once daily, and increased to 3 mg once daily 2 months later, resulted in a worsening of disease activity (DAS20 = 11) and increasing of the IFN signature to 30.5, measured 4 months after the start of baricitinib treatment. For this reason, treatment with baricitinib was ended.

**Figure 1:**
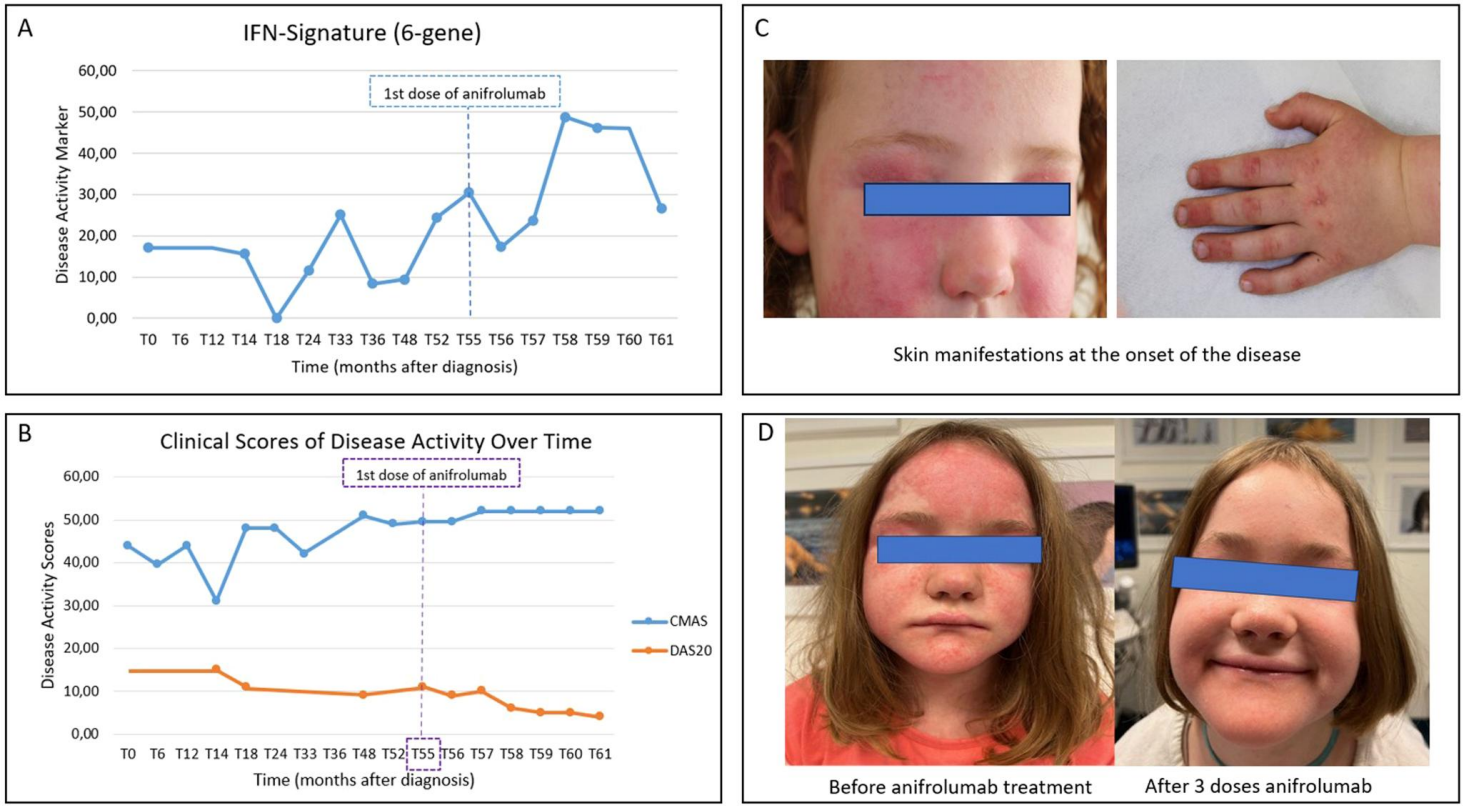
(A) The change in IFN signature (the whole blood expression of six type 1 IFN-stimulated genes measured by real-time PCR: *IFI27*, *IFI44L*, *IFIT1*, *ISG15*, *RSAD2* and *SIGLEC1*; normal <6.0; moderate activity 20.1–50.0) under treatment. **(B)** The change and improvement in CMAS (maximum 52) and DAS20 (worst 20) over the observation period after diagnosis. **(C)** Skin manifestations at onset of the disease. **(D)** Cutaneous improvement after three doses of anifrolumab

Off-label anifrolumab (150 mg/month) was started 4.5 years after the initial diagnosis. Methotrexate (12.5 mg once weekly) and oral prednisolone (3 mg once daily during the first 2 months of treatment and reduced gradually over the following months with a dose of 1 mg every second day after 6 months of treatment with anifrolumab) were continued. All the medications used throughout the course of the disease, including the oral prednisolone dose, are shown in Supplementary Fig. S1. Disease activity improved after a single dose of anifrolumab (DAS20 = 9) and the IFN signature decreased to 17.3 (Fig. 1A, 1B and Supplementary Table S1). The IFN signature values and the values of the individual IFN-regulated genes throughout the course of the disease are shown in Supplementary Table S1. Before the fifth dose, DAS20 improved further to 5 (Fig. 1B). At this time, the vasculitic skin lesions, Gottron’s papules and skin erythema were no longer present and only minimal nailfold erythema remained (Fig. 1D). Furthermore, there were no signs of ongoing myositis and the CMAS remained stable at the highest possible value of 52. No new calcinosis cutis lesions developed, while existing lesions appeared to be healing after spontaneous draining. The IFN signature remained moderately elevated (25.7) after the sixth dose of anifrolumab (Fig. 1A and Supplementary Table S1).

No safety or adverse events were reported and no infections occurred over 7 months of treatment.

In conclusion, skin involvement of JDM rapidly improved in this young patient with refractory disease after just two doses of anifrolumab (150 mg/dose). The IFN signature remained moderately elevated after six doses despite clinical improvement. Calcinosis cutis remained stable. No safety or adverse events occurred.

This case illustrates the potential for anifrolumab to provide additive value to existing treatment strategies, including the use of newer Janus kinase inhibitors, to improve the future treatment of refractory JDM.

Larger prospective studies are required to confirm efficacy and safety in the paediatric population. In addition, longer-term follow-up is required for this promising therapy for JDM to determine the effect of anifrolumab on the course of calcinosis cutis lesions and association with IFN signature.

## Supplementary Material

rkag017_Supplementary_Data

## Data Availability

Data supporting this case report are included in the article.
