# Supplementary material for: Novel use of anifrolumab in a young patient with Jo-1-positive juvenile dermatomyositis
Source: Rheumatol Adv Pract. 2026 Mar 5;10(2):rkag017. doi: 10.1093/rap/rkag017 (PMC13006197; doi:10.1093/rap/rkag017)
Supplement: rkag017_Supplementary_Data [file rkag017_supplementary_data.zip › Supplementary_Data.pdf]

Supplementary material

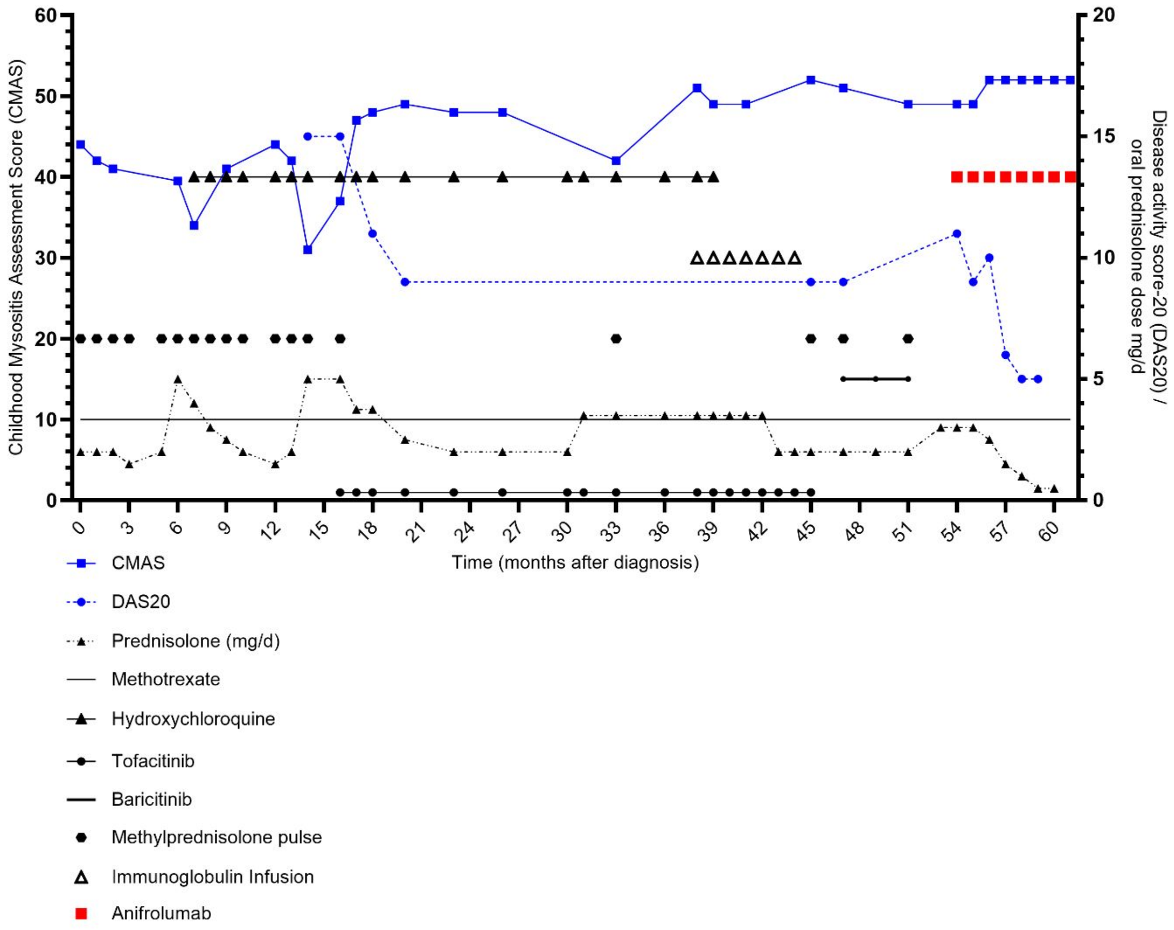

Only

Supplementary Figure S1. Summary of disease activity scores and medication use over the disease course. Disease activity (CMAS, Childhood Myositis Assessment Score, left y axis and DAS-20, Disease Activity Score-20, right axis) over the course of disease from diagnosis to present is shown. Medication use over the disease course is also plotted, with disconnected symbols indicating single-dose intravenous therapies and connected symbols indicating continuous oral or subcutaneous therapies. Additionally, the daily prednisolone dose is indicated, plotted on the right y-axis.

Alt text: Graphical summary of all medications used, as well as the changes in two disease activity scores (CMAS and DAS20) over a 61-month observation period after diagnosis.

Supplementary Table S1: Type 1 Interferon (IFN)-signature calculated as a median of the whole blood expression of six type 1 IFN-stimulated genes: IFI27, IFI44L, IFIT1, ISG15, RSAD2, SIGLEC1 (normal range <6.0). The IFN-signatures under anifrolumab treatment are indicated with an asterisk. Anifrolumab treatment began 55 months after diagnosis (T55).

Time since diagnosis (months)	IFN-Signature	ISG15	RSAD2	IFIT1	IFI44L	IFI27	SIGLEC1
T0	**17,1**	48,0	19,9	6,1	14,2	1436,5	5,2
T3	**26,6**	41,6	33,3	7,6	19,9	2620,9	6,2
T9	**14,1**	27,6	20,6	4,8	7,6	2446,8	2,5
T14	**15,6**	64,3	12,4	7,7	17,9	3273,6	13,4
T18	**23,7**	41,6	32,8	11,3	14,6	969,3	4,5
T24	**11,5**	46,5	8,0	5,8	14,5	56,0	8,5
T30	**48,4**	49,4	47,4	41,9	52,8	58,7	7,5
T33	**25,1**	32,0	26,2	22,5	83,5	24,0	4,4
T36	**8,1**	20,7	7,9	3,3	8,2	49,1	4,7
T42	**32,9**	29,7	42,4	14,9	36,1	63,2	5,8
T45	**13,5**	14,7	12,3	10,7	16,1	16,7	5,5
T48	**9,4**	33,1	6,9	5,0	11,9	43,9	3,4
T52	**24,5**	12,3	65,8	25,4	23,7	77,7	3,5
T55	**30,5***	13,3	37,5	23,5	41,1	79,1	6,5
T56	**17,3***	36,5	15,1	12,9	18,2	133,9	16,5
T57	**23,7***	20,2	24,6	22,8	76,0	50,7	4,4
T58	**48,8***	36,8	60,9	28,6	97,5	132,7	15,2
T59	**46,2***	34,5	57,9	31,3	97,7	203,0	9,0
T61	**25,7***	34,6	16,7	16,8	43,9	114,4	3,0

For Review Only
